# Upregulation of TTYH3 by lncRNA LUCAT1 through interacting with ALYREF facilitates the metastasis in non-small cell lung cancer

**DOI:** 10.1080/15384047.2025.2464966

**Published:** 2025-02-10

**Authors:** Fang Fang, Mei Zhao, Jinming Meng, Jiaqi He, Chunlei Yang, Changhong Wang, Jiaxiao Wang, Sheng Xie, Xiaowei Jin, Wei Shi

**Affiliations:** a Department of OncologyII, The First Affiliated Hospital of Guangxi University of Chinese Medicine, Nanning, Guangxi, P. R. China; b Department of Respiratory and Critical Care Medicine, The First Affiliated Hospital of Guangxi University of Chinese Medicine, Nanning, Guangxi, P. R. China; c Department of Chinese Internal Medicine, Guangxi University of Chinese Medicine, Nanning, Guangxi, P. R. China; d Preventive Treatment of Disease Center, The First Affiliated Hospital of Guangxi University of Chinese Medicine, Nanning, Guangxi, P. R. China; e Department of Integrated TCM & Western Medicine, Yunnan Cancer Hospital & The Third Affiliated Hospital of Kunming Medical University, Kunming, Yunnan, P. R. China

**Keywords:** Non-small cell lung cancer, metastasis, TTYH3, lncRNA LUCAT1, ALYREF

## Abstract

Metastasis is the predominant culprit of cancer-associated mortality in non-small cell lung cancer (NSCLC). Tweety homolog 3 (TTYH3) reportedly functions vitally in the development of diverse cancers, including NSCLC; nevertheless, its role in NSCLC metastasis remains ambiguous. Quantitative reverse transcription polymerase chain reaction (qRT-PCR) and western blot were initially employed to detect TTYH3 expression in NSCLC and normal lung epithelial cells. Subsequently, A549 and NCI-H1650 cells were chosen as NSCLC models in vitro and transfected with short hairpin RNAs (sh-TTYH3, sh-LUCAT1, and sh-ALYREF) or overexpression plasmids (oe-ALYREF and oe-TTYH3). Transwell assays were used for migrative and invasive tests. Epithelial mesenchymal transformation (EMT)-related proteins (E-cadherin, N-cadherin, Vimentin, and Snail) were measured by western blot. A mouse lung metastasis model was built to define the function of TTYH3 in NSCLC metastasis, followed by hematoxylin-eosin staining. RNA pull-down, RNA immunoprecipitation, qRT-PCR, western blot, and actinomycin D assays were adopted to determine the relationships among LUCAT1, ALYREF, and TTYH3. TTYH3 was highly expressed in NSCLC cells relative to normal lung cells. Functionally, TTYH3 knockdown restrained NSCLC migration, invasion, EMT, and metastasis. Mechanistic experiments demonstrated that LUCAT1 bound to ALYREF. After LUCAT1 knockdown, TTYH3 expression and mRNA stability were reduced, which was reversed by ALYREF overexpression. Furthermore, ALYREF overexpression counteracted the inhibitory effects of LUCAT1 knockdown on NSCLC cell migration, invasion, and EMT. TTYH3 overexpression eliminated the suppressive functions of ALYREF downregulation in NSCLC progression. LUCAT1 promotes TTYH3 expression via interacting with ALYREF, thereby facilitating NSCLC migration, invasion, and EMT.

## 1. Introduction

Among all malignancies globally, lung cancer ranks highest in terms of mortality and incidence rates.^1^ Non-small cell lung cancer (NSCLC) is the predominant subtype of lung cancer, constituting roughly 85% of all cases.^2^ Despite the advancements in the treatment, the 5-year survival rate remains dismally low (10%-20%).^3^ The dim prognosis and treatment challenges are largely ascribed to the propensity for metastasis of NSCLC.^4^ Consequently, a better knowledge of the mechanisms driving NSCLC metastasis is essential for developing more effective therapeutic approaches.

As the third member of the Tweety homolog (TTYH) family in mammals, TTYH3 encodes the TTYH3 protein which generates calcium-activated chloride channels.^5^ TTYH3 is primarily produced in excitable tissues like the brain, heart, and skeletal muscle.^6^ Recent investigations have underscored the crucial implications of TTYH3 for tumorigenesis and metastasis.^7–9^ Furthermore, high TTYH3 expression correlates with the poor prognosis of multiple tumors, such as ovarian cancer,^10^ hepatocellular carcinoma,^8^ bladder cancer,^7^ and colorectal cancer.^9^ In lung cancer, TTYH3 is overexpressed and its knockdown can restrain tumor cell proliferation.^11^ Furthermore, high TTYH3 expression demonstrated worse immunotherapy response and shorter survival in patients with lung cancer following immune checkpoint blockade treatment.^11^ Nevertheless, the role of TTYH3 in NSCLC metastasis remains undefined.

Long non-coding RNAs (lncRNAs) are an extremely heterogeneous set of transcripts that comprise diverse RNA species with a length greater than 200 nucleotides.^12^ They lack obvious protein-coding capacity and modulate gene expression through various pathways.^12^ Till now, lncRNAs have been demonstrated to have vital implications for cancer occurrence and metastasis.^13^ The most extensively studied mechanism involving lncRNAs over the past decade has been the microRNA-mediated interaction with mRNAs. However, recent research has increasingly focused on the interaction between lncRNAs and RNA-binding proteins (RBPs).^14^ Through the lncRNA-RBP interaction network, lncRNAs can regulate the expression and stability of the target mRNAs, which play significant roles in cancer onset and development.^15^ In particular, the lung cancer-related transcript 1 (LUCAT1) is a novel lncRNA that is implicated in smoking-related lung cancer.^16^ A previous study demonstrated that LUCAT1 was highly expressed in NSCLC tissues and was associated with the poor prognosis of NSCLC.^17^ Moreover, emerging evidence demonstrates that LUCAT1 serves as a driver of NSCLC migration, invasion, and metastasis.^18^

Studies demonstrate that RBP Aly/REF export factor (ALYREF) is involved in tumor cell proliferation and metastasis.^19–21^ Notably, ALYREF has been indicated to be upregulated in NSCLC and exert tumorigenic functions in this cancer.^22^ Here, we used ENCORI database to predict the upstream molecules of TTYH3 and found that ALYREF was a likely target of LUCAT1 and it could target TTYH3. Hence, we supposed that LUCAT1 might modulate TTYH3 expression by interacting with ALYREF, thus facilitating NSCLC metastasis.

In this context, this work was designed to uncover the role of TTYH3 in NSCLC metastasis. Mechanically, we delved into the crosstalk among TTYH3, ALYREF, and LUCAT1. Our observations might offer fresh ideas about the molecular mechanism of NSCLC metastasis and furnish therapeutic strategies to improve the clinical outcomes of patients with NSCLC.

## 2. Methods

### 2.1. Cell culture and transfection

Human normal lung epithelial cells (BEAS-2B) and NSCLC cells (A549, NCI-H1299, NCI-H1650, and HCC827) were supplied by American Type Cell Culture (Rockville, MD, USA). BEAS-2B and A549 cells were cultured in DMEM (Gibco, MD, USA). NCI-H1299, NCI-H1650, and HCC827 cells were cultivated in RPMI-1640 medium. All mediums contained 10% fetal bovine serum (FBS, Gibco, Grand Island, NY, USA) and 1% penicillin-streptomycin (Logan, UT, USA) and maintained at 37°C with 5% CO_2_.

The short hairpin RNAs (shRNAs) of TTYH3 (sh-TTYH3–1, sh-TTYH3–2, and sh-TTYH3–3), LUCAT1 (sh-LUCAT1–1, sh-LUCAT1–2, and sh-LUCAT1–3), ALYREF (sh-ALYREF-1, sh-ALYREF-2, and sh- ALYREF-3), the overexpression plasmids of ALYREF (oe-ALYREF) and TTYH3 (oe-TTYH3), and their negative controls (sh-NC and oe-NC) were acquired from GenePharma (Shanghai, China). Cell transfection was achieved via Lipofectamine 3000 (Invitrogen, CA, USA) for 48 h. Quantitative reverse transcription polymerase chain reaction (qRT-PCR) and western blot were applied to identify transfection efficiency.

### 2.2. Quantitative reverse transcription polymerase chain reaction (qRT-RCR)

Total RNA was extracted from cells or tissues using TRIzol reagent (Invitrogen). FastKing-RT SuperMix (Tiangen, Beijing, China) was employed for reverse transcription. Next, RT-RCR was carried out on the CFX Connect RT-PCR detection system (Bio-Rad, CA, USA) through SYBR Green PCR Master Mix (Thermo Fisher Scientific, MA, USA). The 2^−ΔΔCT^ method was used to quantify gene expression with GAPDH as the internal control. The primers are displayed in Table 1.

**Table 1.** Primers for qRT-PCR.

Gene	Primer sequences (5’ to 3’)
TTYH3	Forward: CAGAGTGGGGAGGGGAGT Reverse: CTGGGCAGGTTGGCTGT
LUCAT1	Forward: GCTCGGATTGCCTTAGACAG Reverse: TGCCAAGGTCCCATAAGAGT
ALYREF	Forward: GCAGGCCAAAACAACTTCCC Reverse: AGTTCCTGAATATCGGCGTCT
GAPDH	Forward: GAGTCAACGGATTTGGTCGT Reverse: GACAAGCTTCCCGTTCTCAG

### 2.3. Western blot

Total protein was separated using radioimmunoprecipitation assay lysis buffer (Beyotime, Shanghai, China). A bicinchoninic acid kit (Solarbio, Beijing, China) was utilized for protein quantification. Protein separation was achieved via 10% sodium dodecyl sulfate-polyacrylamide gel electrophoresis (Beyotime). Afterward, proteins were migrated to polyvinylidene difluoride membranes (Beyotime) and sealed with 5% nonfat milk (Beyotime). After overnight incubation at 4°C with primary antibodies, membranes underwent incubation with Goat anti-Rabbit IgG H&L (HRP) (1: 2,000; Abcam, Cambridge, UK, #ab6702). Finally, enhanced chemiluminescence kits (APPLYGEN, Beijing, China) were employed for membrane visualization. Below are the primary antibodies used: TTYH3 (1: 5,000; Proteintech, IL, USA, #24707–1-AP), E-cadherin (1: 20000; Proteintech, #20874–1-AP), N-cadherin (1:5,000; Abcam, #ab76011), Vimentin (1: 2,000; Abcam, #ab92547), Snail (1: 1,000; Abclonal, Wuhan, China, #A5243), ALYREF (1:2,000; Abcam, #ab202894), and GAPDH (1: 10000; Abcam, #ab181602).

### 2.4. Transwell assay

A549 and NCI-H1650 cells (1 × 10^5^) were plated into the upper chamber of a 24-well plate with or without pre-coated Matrigel. The lower chamber was added with RPMI-1640 medium containing 10% FBS. After 24-h incubation, the cells in the lower chamber underwent fixation with 4% paraformaldehyde (30 min), then 0.1% crystal violet staining (20 min). Ultimately, the cells were photographed by a microscope (DMi3000 B, Leica, Wetzlar, Germany), and cell migration or invasion was analyzed.

### 2.5. RNA pull-down assay

Labeling of LUCAT1 or TTYH3 was achieved using biotin (Roche, Basel, Switzerland), and Biotin RNA Labeling Mix (Roche) along with T7 RNA polymerase (Roche) was utilized for transcribing. Subsequently, a mixture of lysis buffer and 80 U/mL RNasin (Promega, WI, USA) was used to lyse A549 and NCI-H1650 cells. Cell lysates (2 μg) was incubated with 100 pmol biotinylated RNA at 4°C for 1 h. Then, the streptavidin‐coupled agarose beads (Invitrogen) were employed to separate the RNA-protein complex. The isolated proteins were determined through western blot.

### 2.6. RNA immunoprecipitation (RIP) assay

A549 and NCI-H1650 cell lysates were obtained using a complete RIP lysis buffer (Millipore, MA, USA) and subsequently incubated with IgG (1:50; Abcam, #ab172730) and ALYREF (1:40; Abcam, #ab202894) antibodies at 4°C overnight. After RNA purification, qRT-PCR was carried out for the analysis of the immunoprecipitated RNA.

### 2.7. Actinomycin D assay

A549 and NCI-H1650 cells were administrated with 2 μg/mL actinomycin D for different periods (0, 3, and 6 h). Total RNA was then extracted, and changes in TTYH3 mRNA stability were defined by qRT-PCR.

### 2.8. Animal experiments

Eighteen specific pathogen-free male BALB/c nude mice (4 weeks old) were sourced by SLACOM (Shanghai, China). Mice were bred under the following conditions: a 12-h light/dark cycle; 22 ± 3°C; 45 ± 10% humidity. All mice were allowed *ad libitum* food and water.

After 7-day acclimatization, the mice were randomly split into sh-NC and sh-TTYH3–1 groups (*n* = 6 mice/group). As per the grouping, mice were tail vein injected with A549 cells (2 × 10^6^) transfected with sh-NC or sh-TTYH3–1. Eight weeks later, all mice were euthanized via cervical dislocation.^23^ The lung tissues were collected for subsequent analyses.

### 2.9. Hematoxylin-eosin (HE) staining

By the supplier’s directions, HE staining kits (Beyotime) were employed for histopathological tests. The mouse lung tissues were fixed in 4% paraformaldehyde for 24 h and dehydrated with graded ethanol concentrations. Subsequently, the tissues were clarified with xylene, embedded in paraffin, sliced at 5 μm, dewaxed, and hydrated. After staining, the section images were captured by a microscope (DMi3000 B, Leica), and the metastatic lung nodules were quantified.

### 2.10. Statistical analysis

Data were displayed as means ± standard deviation and analyzed via GraphPad Prism 8.0 (GraphPad, La Jolla, CA, USA). Student’s t-test or one-way analysis of variance was employed to identify group discrepancy. *p* < .05 denoted a significant variation.

## 3. Results

### 3.1. TTYH3 expression is upregulated in NSCLC cells and facilitates tumor cell migration, invasion, and EMT

**Figure 1.**
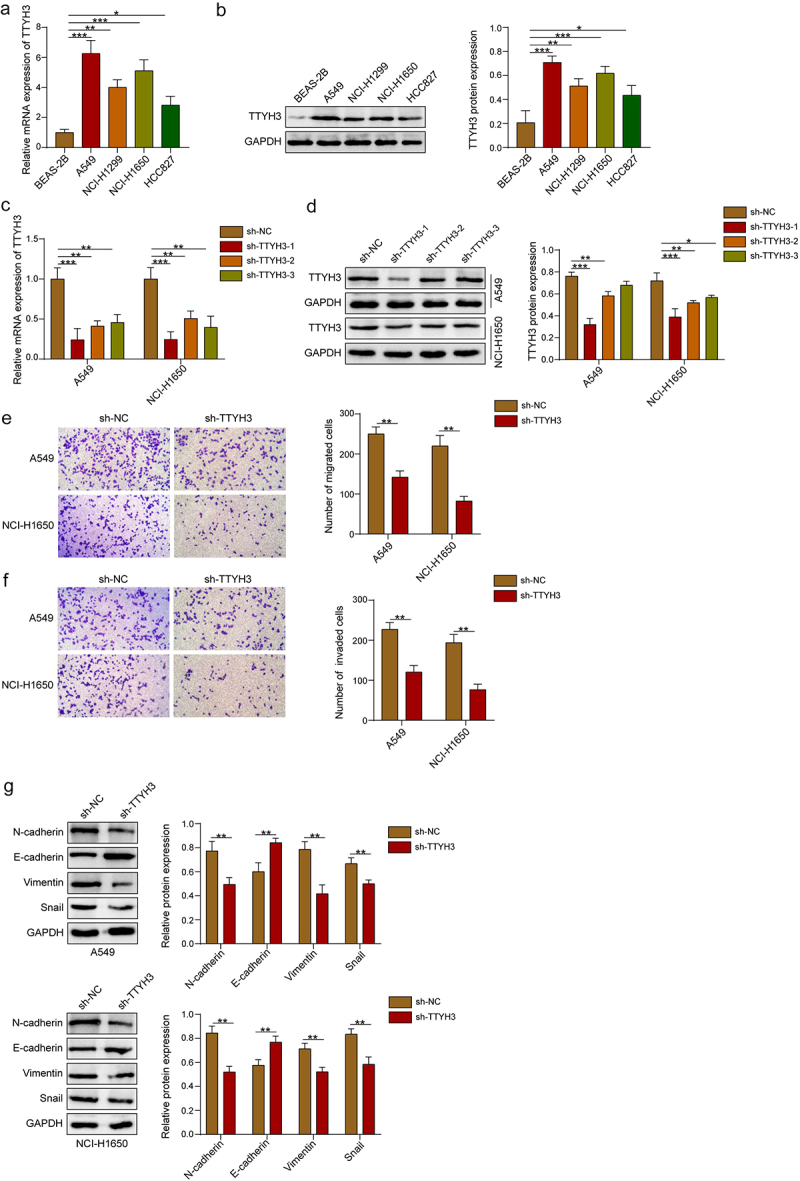
TTYH3 knockdown suppresses the migration, invasion, and EMT of NSCLC cells. (a-b) qRT-PCR and western blot were used to detect the expression levels of TTYH3 in human normal lung epithelial cell line (BEAS-2B) and NSCLC cell lines (A549, NCI-H1299, NCI-H1650, and HCC827). (c-d) qRT-PCR and western blot were employed to verify the knockdown efficacy of TTYH3 in A549 and NCI-H1650 cells and chose the optimal sh-TTYH3 sequence for further experiments. (e-f) Transwell assays were performed to assess the migration and invasion of A549 and NCI-H1650 cells. (g) Western blot was used to evaluate the expression levels of emt-related proteins (E-cadherin, N-cadherin, Vimentin, and Snail) in A549 and NCI-H1650 cells. Data were exhibited as means ± standard deviation. *p < .05, **p < .01, ***p < .001. TTYH3, Tweety homolog 3; EMT, epithelial-mesenchymal transition; NSCLC, non-small cell lung cancer; qRT-PCR, quantitative reverse transcription polymerase chain reaction.

TTYH3 expression was initially detected in human normal lung epithelial cells (BEAS-2B) and NSCLC cells (A549, NCI-H1299, NCI-H1650, and HCC827). We observed markedly higher TTYH3 expression in NSCLC cells than BEAS-2B cells (Figure 1a,b). Since A549 and NCI-H1650 cells exhibited the highest TTYH3 expression among the four NSCLC cell lines, they were chosen for follow-up assays.

Next, three shRNAs of TTYH3 (sh-TTYH3–1, sh-TTYH3–2, and sh-TTYH3–3) were applied to establish TTYH3-deficient NSCLC cells. All the shRNAs significantly reduced TTYH3 expression in A549 and NCI-H1650 cells (Figure 1c,d). Given the optimal knockdown effectiveness, sh-TTYH3–1 was selected for subsequent experiments. Transwell assays displayed that TTYH3 knockdown observably restrained NSCLC cell migration and invasion (Figure 1e,f). Also, we analyzed the expressions of EMT-related proteins (E-cadherin, N-cadherin, Vimentin, and Snail) as epithelial-mesenchymal transition (EMT) is indispensable for tumor progression and metastasis.^24^ Here, epithelial indicator E-cadherin was dramatically augmented in NSCLC cells following TTYH3 knockdown, while mesenchymal indicators N-cadherin, Vimentin, and Snail were reduced (Figure 1g).

### 3.2. Knockdown of TTYH3 suppresses NSCLC metastasis in vivo

**Figure 2.**
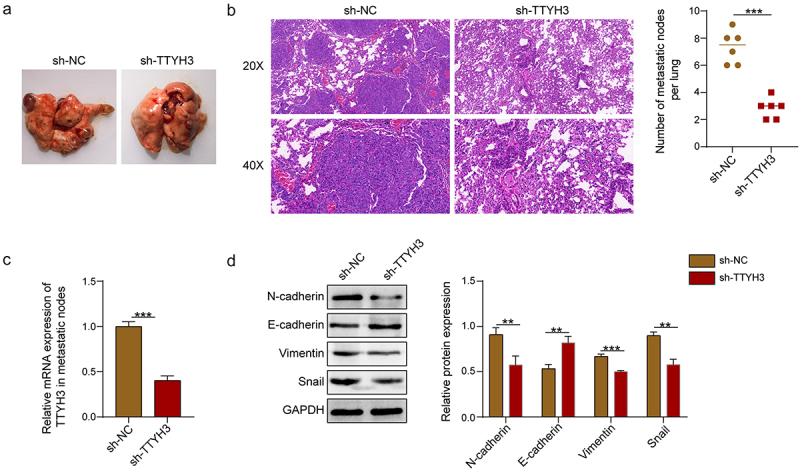
Downregulation of TTYH3 inhibits NSCLC metastasis *in vivo*. (a) Representative images of nude mouse lung tissues. (b) Representative images of lung metastatic tumor foci after HE staining and statistical chart of the number of metastatic nodules. (c) qRT-PCR was used to detect TTYH3 expression in lung metastatic nodules. (d) Western blot was employed to evaluate the expression levels of E-cadherin, N-cadherin, Vimentin, and Snail in lung metastatic nodules. A549 cells (2 × 10^6^) transfected with sh-nc or sh-TTYH3–1 were tail vein injected into BALB/c nude mice (*n* = 6 mice/group). After 8 weeks, all mice were sacrificed. Data were exhibited as means ± standard deviation. **p < .01, ***p < .001. TTYH3, Tweety homolog 3; NSCLC, non-small cell lung cancer; qRT-pcr, quantitative reverse transcription polymerase chain reaction.

To ascertain the role of TTYH3 in NSCLC metastasis, a mouse lung metastasis model was built via tail vein injecting A549 cells transfected with sh-NC or sh-TTYH3 into BALB/c nude mice. After eight weeks, evident lung metastasis was observed in both sh-NC and sh-TTYH3 groups (Figure 2a). HE staining identified the nodules as metastatic tumor foci, and there was a marked reduction in the nodule number in the sh-TTYH3 group in comparison with the sh-NC group (Figure 2b). Additionally, TTYH3 mRNA expression was substantially lowered in the lung metastatic nodules following TTYH3 knockdown (Figure 2c). Regarding EMT, TTYH3 downregulation conduced to a notable elevation of E-cadherin expression and decreases in N-cadherin, Vimentin, and Snail in the lung metastatic nodules (Figure 2d). These findings demonstrated that TTYH3 facilitated tumor metastasis in NSCLC.

### 3.3. LUCAT1 interacts with ALYREF to enhance TTYH3 expression and mRNA stability

**Figure 3.**
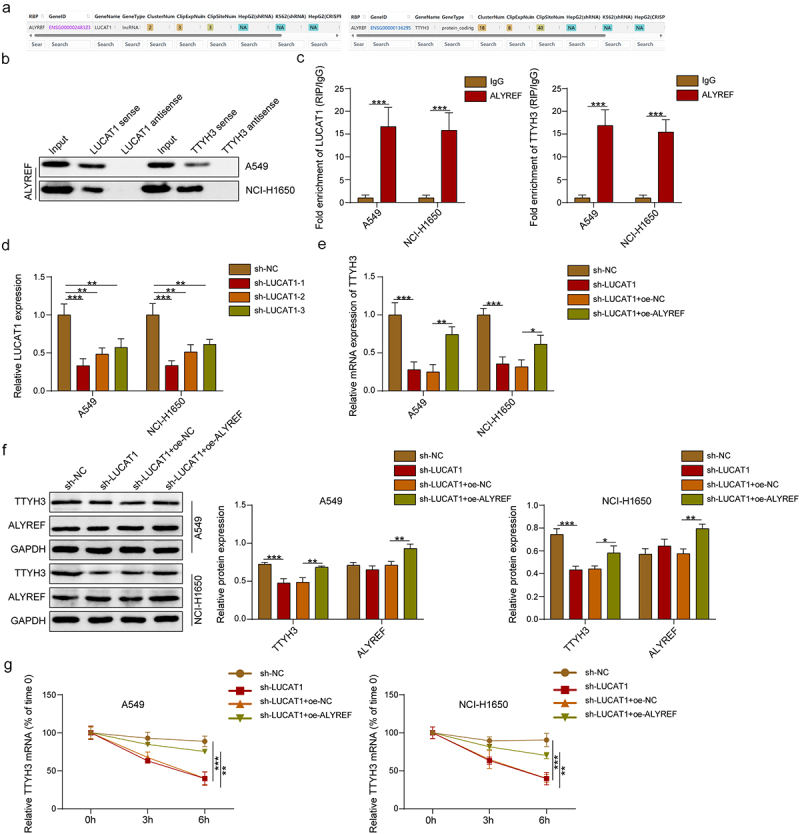
The binding of LUCAT1 to ALYREF enhances TTYH3 expression and mRNA stability in NSCLC cells. (a) The binding sites between ALYREF and LUCAT1 or TTYH3 were predicted using ENCORI database. (b-c) RNA pull-down and RIP assays were conducted to validate the binding of ALYREF with LUCAT1 and TTYH3 in A549 and NCI-H1650 cells. (d) qRT-PCR was used to validate the knockdown efficacy of LUCAT1 in A549 and NCI-H1650 cells and select the optimal sh-LUCAT1 sequence for further assays. (e) qRT-PCR was conducted to assess TTYH3 expression in A549 and NCI-H1650 cells. (f) Western blot was employed to evaluate the protein expression levels of TTYH3 and ALYREF in A549 and NCI-H1650 cells. (g) The expression levels of TTYH3 were measured in A549 and NCI-H1650 cells at 0, 3, and 6 h following actinomycin D treatment using qRT-PCR. Data were exhibited as means ± standard deviation. *p < .05, **p < .01, ***p < .001. LUCAT1, lung cancer-related transcript 1; ALYREF, Aly/ref export factor; TTYH3, Tweety homolog 3; NSCLC, non-small cell lung cancer; RIP, RNA immunoprecipitation; qRT-pcr, quantitative reverse transcription polymerase chain reaction.

Subsequently, we investigated the upstream regulatory mechanism of TTYH3 driving NSCLC metastasis. In NSCLC, lncRNAs can regulate the expression and stability of the target genes via binding to RBPs, thereby affecting cancer development.^15^ Moreover, previous studies demonstrated that lncRNA LUCAT1^17^ and RBP ALYREF^22^ were highly expressed in NSCLC. Using ENCORI database (https://rnasysu.com/encori/.), we observed that ALYREF could bind to LUCAT1 and TTYH3 (Figure 3a). According to RNA pull-down (Figure 3b) and RIP (Figure 3c) assays, we observed that LUCAT1 and TTYH3 were enriched in ALYREF promoter in NSCLC cells. This demonstrated that ALYREF could bind to LUCAT1 and TTYH3, suggesting that LUCAT1 might regulate TTYH3 mRNA stability and expression by interacting with ALYREF. Then, we established LUCAT1-knockdown and ALYREF-overexpressing NSCLC cell lines (A549 and NCI-H1650). We utilized three shRNAs of LUCAT1 (sh-LUCAT1–1, sh-LUCAT1–2, and sh-LUCAT1–3) and chose sh-LUCAT1–1 for further assays given its optimal knockdown effectiveness (Figure 3d). Here, LUCAT1 knockdown led to notable decreases in TTYH3 expression in NSCLC cells, which was reversed by ALYREF overexpression (Figure 3e,f). Additionally, LUCAT1 knockdown observably reduced TTYH3 mRNA stability in NSCLC cells when treated with actinomycin D, which was restored by ALYREF overexpression (Figure 3g). The above data indicated that the binding of LUCAT1 to ALYREF could enhance TTYH3 stability and expression in NSCLC cells.

### 3.4. LUCAT1/ALYREF axis facilitates the migration, invasion, and EMT of NSCLC cells

**Figure 4.**
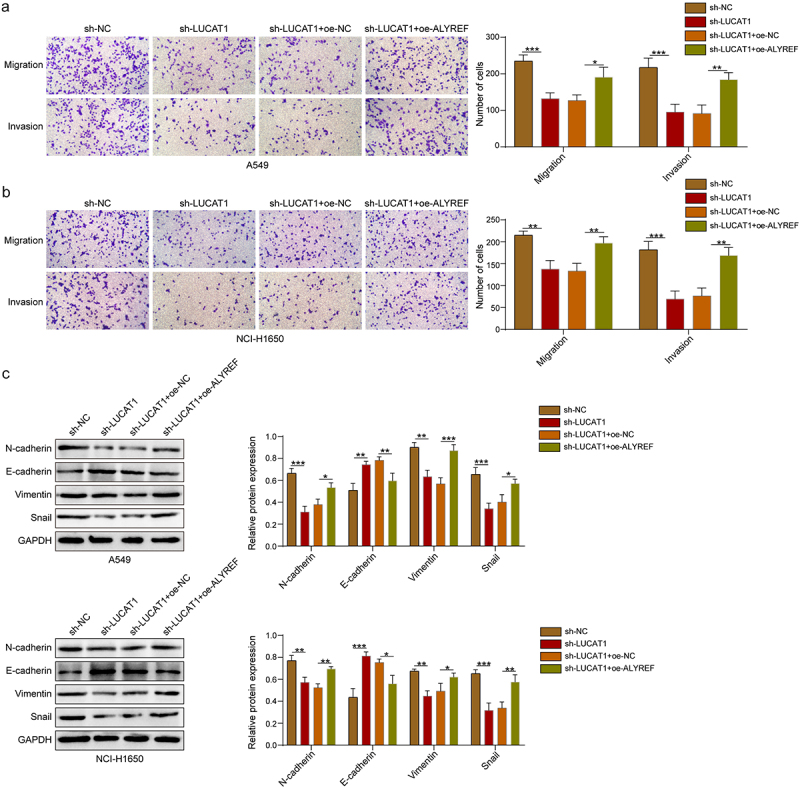
LUCAT1 interacts with ALYREF to facilitate the migration, invasion, and EMT of NSCLC cells. (a-b) Transwell assays were conducted to evaluate the migration and invasion of A549 and NCI-H1650 cells. (c) Western blot was employed to measure the expression levels of E-cadherin, N-cadherin, Vimentin, and Snail in A549 and NCI-H1650 cells. Data were exhibited as means ± standard deviation. *p < .05, **p < .01, ***p < .001. LUCAT1, lung cancer-related transcript 1; ALYREF, Aly/ref export factor; EMT, epithelial-mesenchymal transition; NSCLC, non-small cell lung cancer.

Functional assays were carried out to further define the impacts of the LUCAT1-ALYREF interaction on NSCLC progression. Our data demonstrated that LUCAT1 deficiency significantly restrained NSCLC cell migration and invasion (Figure 4a-b). Moreover, LUCAT1 knockdown prominently suppressed the EMT of NSCLC cells, as evidenced by elevated E-cadherin expression and reduced N-cadherin, Vimentin, and Snail (Figure 4c). Nevertheless, these effects of LUCAT1 knockdown were undone following ALYREF overexpression (Figure 4a-c).

### 3.5. ALYREF promotes the migration, invasion, and EMT of NSCLC cells through interacting TTYH3

**Figure 5.**
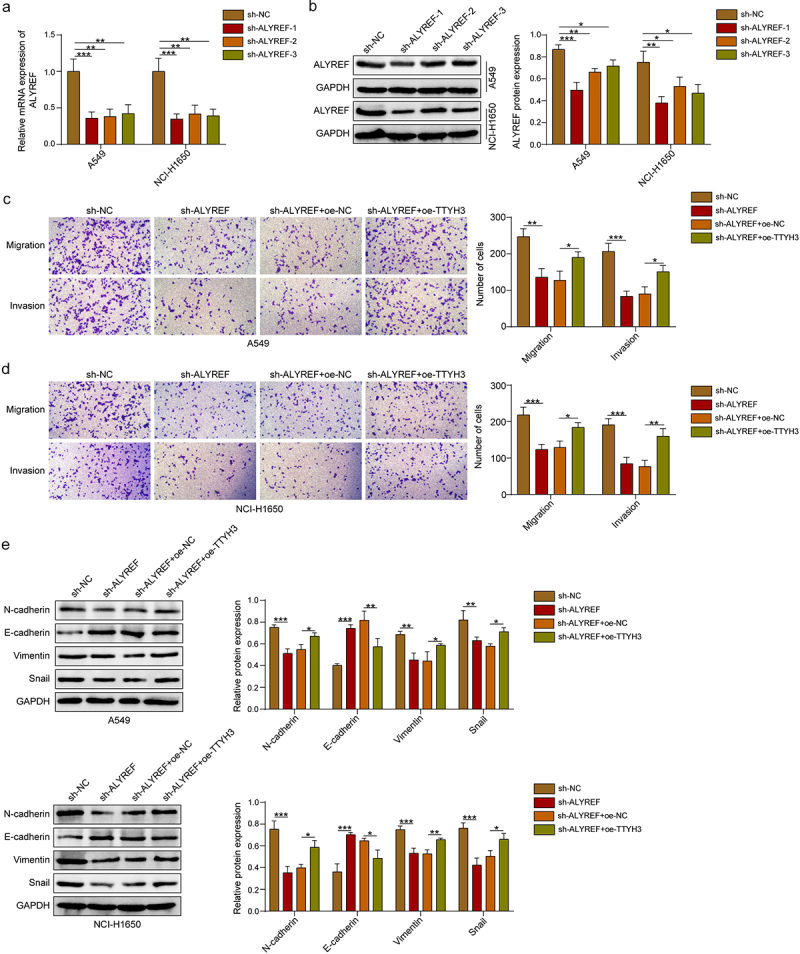
ALYREF/TTYH3 signaling contributes to the malignant progression of NSCLC *in vitro*. (a-b) qRT-PCR and western blot were used to validate the knockdown efficacy of ALYREF in A549 and NCI-H1650 cells and select the optimal sh-alyref sequence for further experiments. (c-d) Transwell assays were conducted to evaluate the migration and invasion of A549 and NCI-H1650 cells. (e) Western blot was employed to detect the expression levels of E-cadherin, N-cadherin, Vimentin, and Snail in A549 and NCI-H1650 cells. Data were exhibited as means ± standard deviation. *p < .05, **p < .01, ***p < .001. ALYREF, Aly/ref export factor; TTYH3, Tweety homolog 3; NSCLC, non-small cell lung cancer; qRT-pcr, quantitative reverse transcription polymerase chain reaction.

To further pinpoint the function of ALYREF in TTYH3-mediated NSCLC progression, we constructed ALYREF-deficient and TTYH3-overexpressing NSCLC cells (A549 and NCI-H1650). Three shRNAs of ALYREF (sh-ALYREF-1, sh-ALYREF-2, and sh-ALYREF-3) were applied. Sh-ALYREF-1 was selected for subsequent analyses for it is superior to the other two in knockdown efficiency (Figure 5a-b). Further functional analyses revealed that ALYREF knockdown significantly impeded NSCLC cell migration, invasion, and EMT (Figure 5c-e). Importantly, the functions of ALYREF downregulation were eliminated by TTYH3 overexpression (Figure 5c-e). Taken together, these observations indicated that ALYREF/TTYH3 signaling facilitated the malignant phenotypes of NSCLC cells.

## 4. Discussion

Metastasis remains the primary cause of cancer-induced death in NSCLC,^3^ making it urgent to further understand the molecular mechanism to develop more effective therapies. Recent research has identified chloride channels as promising targets for therapeutic interventions in cancer metastasis.^25^ TTYH3, a large conductance chloride channel, plays crucial roles in the development of diverse cancers^7–9,26^ and has reportedly been highly expressed in NSCLC.^11^ Hence, we delved into the function and mechanism of TTYH3 in NSCLC metastasis. Here, we found that TTYH3 promoted NSCLC cell migration, invasion, EMT, and tumor metastasis. In terms of mechanism, the oncogenicity of TTYH3 in NSCLC can be ascribed to the interplay between lncRNA LUCAT1 and ALYREF.

TTYH3 encodes high-conductivity chloride ion channels activated by Ca.^2+27^ Emerging evidence indicates that TTYH3 is aberrantly expressed in diverse cancers and functions substantially in tumor onset and progression.^7–9,26^ For instance, TTYH3 is upregulated in bladder cancer^7^ and hepatocellular carcinoma,^8^ the knockdown of which could suppress tumor progression. Notably, TTYH3 expression is elevated in lung cancer tissues and correlates with the poor prognosis.^11^ Consistently, our investigations also demonstrated notable upregulation of TTYH3 expression in NSCLC cells in contrast with normal lung cells. Although its role as an oncogene in lung cancer is established,^11^ the implication of TTYH3 in NSCLC metastasis was previously uncertain. EMT is linked to the malignant characteristics of cancer cells during tumor progression and metastasis like migration and invasion.^24^ Hence, we explored the function of TTYH3 in EMT by evaluating the expressions of EMT-associated proteins (E-cadherin, N-cadherin, Vimentin, and Snail). Our data indicated that TTYH3 deficiency could inhibit NSCLC cell migration and invasion. Moreover, epithelial indicator E-cadherin was upregulated in NSCLC cells following TTYH3 knockdown, whereas mesenchymal indicators N-cadherin, Vimentin, and Snail were downregulated. This suggested a favorable impact of TTYH3 downregulation on suppressing EMT in NSCLC. Importantly, TTYH3 knockdown conduced to diminished lung metastatic nodules in the mouse model and restrained EMT in the metastatic nodules. The functions of TTYH3 in NSCLC migration, invasion, EMT, and metastasis were consistent with those in hepatocellular carcinoma.^8^ These observations indicated that TTYH3 exacerbated NSCLC progression.

Subsequently, we further investigated the regulatory mechanism of TTYH3 in NSCLC. Lately, the mechanism by which cancer-associated genes are regulated through the binding of lncRNAs with RBPs has brought about widespread attention.^14^ In NSCLC, lncRNAs can modulate the expression and stability of target mRNAs via combining RBPs and forming ribonucleoprotein complexes.^15^ For example, the binding of lncRNA LCAT1 to IGF2BP2 could facilitate CDC6 mRNA stability, thereby promoting NSCLC cell proliferation and migration.^28^ Moreover, lncRNA ITGB2-AS1 can interact with FOSL2 to augment NAMPT expression, resulting in NSCLC cisplatin resistance.^29^ Accordingly, we concentrated on the interplay between lncRNA and RNA binding protein to further elucidate the mechanism of TTYH3 promoting NSCLC metastasis.

Intriguingly, we discovered that RBP ALYREF could interact with lncRNA LUCAT1 and TTYH3 in NSCLC cells. LUCAT1 was originally observed in the airways of patients with a smoking history, and thus it is occasionally termed smoke and carcinoma-related lncRNA-1.^30^ Prior data demonstrated that LUCAT1 could foster NSCLC migration, invasion, EMT, and metastasis,^18^ with its overexpression correlating with a worse prognosis.^17^ As the first identified m^5^C reader, ALYREF predominantly binds to the 3’ and 5’ regions of mRNA, implicated in mRNA export, stabilization, and splicing.^31,32^ Recent research has linked ALYREF overexpression to the poor prognosis and progression of various tumors, including bladder cancer,^32^ gastric cancer,^33^ and pancreatic ductal adenocarcinoma.^34^ While both LUCAT1^18^ and ALYREF^22^ have been demonstrated to exert oncogenic effects in NSCLC through the lncRNA-RBP-mRNA mechanism, their crosstalk remains unexplored. To determine whether the LUCAT1-ALYREF interplay mediates TTYH3-mediated NSCLC progression, we used LUCAT1-knockdown, ALYREF-overexpressing, ALYREF-knockdown, and TTYH3-overexpressing NSCLC cells. We observed that LUCAT1 knockdown reduced TTYH3 expression and stability, which was restored following ALYREF overexpression. Further functional assays revealed that LUCAT1 knockdown diminished NSCLC cell migration, invasion, and EMT, conforming to earlier research.^18^ Furthermore, previous animal research indicated the oncogenic effects of LUCAT1 on NSCLC. In the mouse models of NSCLC, LUCAT1 knockdown could suppress tumor growth *in vivo*.^17,35^ Importantly, ALYREF overexpression counteracted the functions of LUCAT1 downregulation in preventing NSCLC progression. On the other hand, we demonstrated inhibitory impacts of ALYREF knockdown on NSCLC cell migration, invasion, and EMT, which were eliminated after TTYH3 overexpression. Collectively, our data suggested that LUCAT1 could enhance TTYH3 stability and expression via interacting with ALYREF, thus facilitating NSCLC progression.

Nonetheless, this work has some deficiencies. The association of TTYH3 expression with NSCLC metastasis remains to be further verified with clinical samples. Besides, we explored the mechanism of TTYH3 promoting NSCLC progression largely at the *in vitro* level without *in vivo* validation. Therefore, additional investigations are required to address these gaps.

## 5. Conclusions

Overall, this research unveiled a crucial contribution of TTYH3 to NSCLC metastasis. Mechanically, LUCAT1 interacted with ALYREF, which enhanced TTYH3 stability and expression, ultimately driving NSCLC progression. These observations introduce a fresh LUCAT1/ALYREF/TTYH3 axis implicated in NSCLC metastasis, highlighting it as a likely curative target for NSCLC.

## Highlights

- TTYH3 is overexpressed in NSCLC cells and promotes NSCLC migration, invasion, EMT, and tumor metastasis.
- LUCAT1 enhances TTYH3 expression in NSCLC cells by interacting with ALYREF.
- The LUCAT1/ALYREF/TTYH3 axis facilitates NSCLC metastasis.

## List of abbreviations

NSCLC: non-small cell lung cancer
TTYH3: Tweety homolog 3
ALYREF: Aly/REF export factor
lncRNA: long non-coding RNA
RBP: RNA-binding protein
LUCAT1: lung cancer-related transcript 1
FBS: fetal bovine serum
shRNA: short hairpin RNA
qRT-PCR: quantitative reverse transcription polymerase chain reaction
RIP: RNA immunoprecipitation
HE: hematoxylin-eosin
EMT: epithelial-mesenchymal transition.

## Data Availability

The datasets used or analyzed during the current study are available from the corresponding author on reasonable request.
